# A Systematic Review of Pharmacokinetic Models of Vancomycin in Adult Patients (2020-2024): Trends, Variability, and Key Covariates

**DOI:** 10.2174/0113892002395979251015105103

**Published:** 2025-11-15

**Authors:** Raquel Fresquet-Molina, María de los Ángeles Allende-Bandres, Mercedes Arenere-Mendoza, Lucía Sopena-Carrera, Irene Navarro-Pardo, Ángela Jimeno-Martin, Tránsito Salvador-Gomez, Manuel Gomez-Barrera, Loreto Sáez-Benito-Suescun, Nuria Berenguer-Torrijo

**Affiliations:** 1 Primary Care Pharmacy Unit, Terres de l'Ebre Primary Care Department, Tortosa, Spain;; 2 Grupo de Investigación de Farmacia Hospitalaria (GIFARH), Instituto de Investigación Sanitaria de Aragón, Zaragoza, Spain;; 3 Pharmacy Department, Hospital Clínico Universitario Lozano Blesa, Zaragoza, Spain;; 4 Grupo de Investigación Clínica en Enfermedades Infecciosas, Instituto de Investigación Sanitaria de Aragón, Zaragoza, Spain;; 5 Faculty of Health Science, San Jorge University, Zaragoza, Spain

**Keywords:** Vancomycin, pharmacokinetics, therapeutic drug monitoring, population models, critical care, covariates

## Abstract

**Introduction:**

This systematic review aimed to identify, evaluate, and critically analyze pharmacokinetic models of vancomycin in adult populations published in PubMed and EMBASE between 2020 and 2024.

**Materials and Methods:**

Twenty-two studies were included, describing 24 models characterized by substantial heterogeneity in terms of study populations, methodological design, and covariate selection. Most models were developed in Asia and focused on hospitalized patients, particularly those in intensive care units (ICUs). Data from 2150 patients were analyzed, with an average of 93 patients per model.

**Results:**

The models demonstrated high variability in pharmacokinetic parameters, such as vancomycin clearance (Cl) and volume of distribution (Vd), influenced by factors, such as renal function, weight, age, and comorbidities. The meta-analysis conducted on clearance and interindividual variability in clearance (IIV Cl) revealed high heterogeneity among the analyzed studies. The average vancomycin clearance was 4.23 L/h, with higher values observed in neurosurgical, oncohematologic patients, and those with increased renal function. The volume of distribution showed greater variability in obese patients and those undergoing continuous renal replacement therapy. Creatinine clearance (ClCr) was identified as a significant covariate in 66% of the models, while weight was significant in 33%. Other important covariates included age, sex, serum creatinine, serum urea, and the hospital admission unit. The meta-analysis of Cl and IIV Cl showed high heterogeneity among the studies, with I^2^ values of 0.83 for Cl and 0.98 for IIV Cl, indicating substantial variability.

**Discussion:**

The limitations of this study included the diversity of the analyzed populations, which made it challenging to assess the model's suitability. While the models showed advances in precision, challenges, such as the lack of external validation and discrepancies in dosing recommendations, remain.

**Conclusion:**

This review paper has highlighted the need to validate models in diverse populations and clinical settings to optimize personalized vancomycin therapy in adults. The findings have highlighted the importance of validating or adapting pharmacokinetic models to the specific characteristics of each hospital population.

## 1 INTRODUCTION

Vancomycin is a glycopeptide antibiotic widely used to treat severe infections caused by gram-positive bacteria, including methicillin-resistant *Staphylococcus aureus* [1-3]. Given its narrow therapeutic index, therapeutic drug monitoring (TDM) is recommended to optimize vancomycin’s efficacy and minimize nephrotoxicity. International guidelines recommend targeting an area under the curve over 24 hours (AUC 24) to a minimum inhibitory concentration (MIC) ratio (AUC/MIC 24) of 400-700 as the primary pharmacokinetic/ pharmacodynamic (PK/PD) goal for therapy [3]. Traditionally, trough concentrations were used as a surrogate for AUC; however, this approach has proven inadequate for accurately estimating AUC. Bayesian-based approaches, which incorporate multiple vancomycin concentration measurements, are now preferred for optimizing AUC estimation and dose individualization [3-6].

Despite these advances, vancomycin pharmacokinetics (PK) exhibit significant variability, influenced by patient-specific factors, such as age, weight, renal function, and comorbidities (interindividual variability), as well as dynamic changes within the same patient over time (intraindividual variability) [6, 7]. Additionally, obesity significantly impacts vancomycin distribution and clearance, necessitating weight-adjusted dosing to optimize therapeutic outcomes [8]. Moreover, models that incorporate specific population characteristics, such as pregnant women or neurosurgical patients, provide improved dosing strategies tailored to these unique groups [9, 10]. Population pharmacokinetic (PopPK) models are an essential tool for understanding these variabilities, enabling the development of personalized dosing regimens.

Two notable reviews have analyzed vancomycin PopPK models to date. Marsot *et al*. [11] reviewed studies up to 2010, reporting variability in model structures and covariate selection. Similarly, Aljutayli *et al*. [12] reviewed articles from 2011 to 2019, identifying further inconsistencies in patient populations and methodological approaches. However, no systematic review has been conducted to synthesize evidence on PopPK models published after 2020, a period marked by an exponential growth in this field. A preliminary search in the PubMed database has shown a substantial increase in vancomycin-related PopPK studies, rising from 163 articles in 2010–2015 to 237 in 2016-2020.

This systematic review aimed to identify, evaluate, and critically analyze pharmacokinetic models of vancomycin in adult populations. By synthesizing evidence from recent research, the review sought to highlight key trends in model design, covariate selection, and methodological approaches, with an emphasis on the validation of pharmacokinetic models.

## 2 MATERIALS AND METHODS

**Fig. (1):**
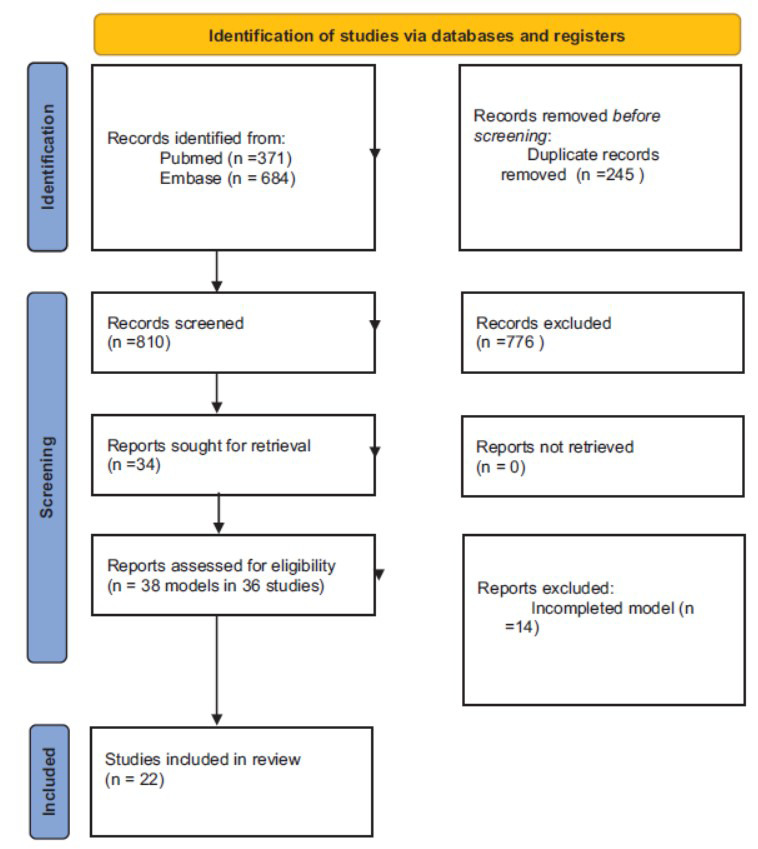
Flow diagram of search results and selection process of the studies. PopPK: population Pharmacokinetic model. PRISMA 2020 flow diagram illustrating the study selection process for the systematic review, detailing the number of records identified, excluded, and ultimately included in the analysis.

This systematic review followed the guidelines outlined in the Preferred Reporting Items for Systematic Reviews and Meta-analyses (PRISMA) statement to ensure methodological rigor and transparency (Fig. **1**). The review protocol was registered in the Open Science Framework.

### 2.1 Search Strategy

A systematic search was conducted in PubMed/MEDLINE, Embase, and Google Scholar. The search terms included ((Vancomycin (MeSH terms)) OR (vancomycin (all fields))) AND ((“Pharmacokinetic model” (all fields)) OR (“Population pharmacokinetics” (all fields)) OR (“Bayes theorem” (MeSH terms)) OR (“Drug monitoring” (MeSH terms))). Filters were applied to include only studies published from January 2020 to February 2024, conducted in humans, and restricted to adult populations (≥18 years). The search was performed by three of the authors (RF, MA, MAA, NB).

### 2.2 Eligibility Criteria

Studies were included if they met the following criteria: 1) original PopPK models of vancomycin published in journals, 2) focus on adult populations (≥18 years), 3) use of compartmental modeling approaches, including one-, two-, or multi-compartment models, and 4) inclusion of key pharmacokinetic parameters, such as Cl, Vd, and covariate relationships.

The exclusion criteria were as follows: 1) studies conducted in pediatric or neonatal populations (<18 years); 2) models developed exclusively *in silico* or using non-compartmental approaches; 3) models developed exclusively for continuous infusion of vancomycin; 4) conference abstracts, review articles, case reports, study protocol or non-peer-reviewed studies; 5) incomplete models lacking essential PK parameters or covariate analysis; and 6) studies involving mixed populations; these studies were only included if results specific to vancomycin were delineated.

### 2.3 Data Collection and Analysis

All retrieved articles were imported into reference management software, and duplicates were removed using Mendeley. Two independent reviewers screened titles and abstracts, analyzed the data, and subsequently extracted relevant information from the articles using a standardized extraction form. Discrepancies were resolved through discussion or consultation with a third reviewer.

The extracted variables included study characteristics, such as the first author, publication year, and country of origin. Population characteristics were also recorded, including sample size, age, sex, body weight, body mass index, renal function parameters (creatinine clearance), and the clinical context. Model characteristics were detailed, encompassing the structural model type (one-, two-, three-compartment), covariates included, pharmacokinetic parameters, and the Bayesian software used for model development. Methodological details were documented as well, including the number of samples per patient, whether steady-state sampling was performed, and the type of validation methods applied (internal *versus* external).

### 2.4 Quality Assessment

Internal validity was analyzed following the guidelines of the Centre for Reviews and Dissemination for conducting systematic reviews in healthcare. The following methodological aspects were selected to assess the quality of the articles included in this review: measurement of variability, baseline comparability, risk of contamination, and blinded assessment. To critically appraise the selected articles, the ClinPK checklist, specifically designed for clinical pharmacokinetic studies, was used. This tool includes 24 items organized into different sections, such as title, abstract, introduction, methods, results, and discussion, ensuring a comprehensive and specific evaluation of methodological quality. This score was developed using Delphi methodology as a “best practice” scale to ensure quality of clinical pharmacokinetic studies.

### 2.5 Data Synthesis

Extracted data were synthesized narratively, focusing on commonalities and differences in model design, covariates, and pharmacokinetic parameters. The mean and standard deviation (SD) values for clearance and volume of distribution were calculated using data reported in the original studies. Trends in covariate selection and structural model choices were analyzed to identify gaps and areas for future research.

The homogeneity and selection bias of the studies included in the systematic review were analyzed to assess the feasibility of conducting a meta-analysis. Homogeneity was determined using the I^2^ heterogeneity index, while selection bias was assessed through Egger's test and the funnel plot. All calculations were performed using SPSS 29.0, licensed to Universidad San Jorge.

## 3 RESULTS

### 3.1 Study Characteristics

Two hundred and thirty-seven articles were identified, from which 22 original studies and 24 PopPK models were selected, as two articles described two models each [10, 13] (Fig. **1**). The populations in which the models were developed varied. Specifically, the studies included 11 models developed in intensive care unit (ICU) patients, 6 in general hospitalized patients, 1 in neurosurgical patients, 3 in hematological patients, 1 in renal impairment patients, and 1 in gynecological patients. Most models were derived from studies conducted in Asia: six in China [10, 14-18], three in Japan [19-21], two in South Korea [22, 23], and one in Thailand [24]. The number of patients per model ranged from 16 to 374 [8, 14] with a mean (SD) of 93.47 (87.33) patients per PopPK model and a total of 2150 patients. The anthropometric and demographic characteristics of the patients are shown in Table **1**.

### 3.2 Model Characteristics

#### 3.2.1 Study Protocol

The median number of blood samples collected per patient was 4 (range 1-9), with a total of 6,843 blood samples across all studies. The mean (SD) number of blood samples per model was 325.8 (286). Prospective studies [8, 16, 22, 23, 25, 26] adhered to a predefined sampling schedule outlined in the study protocol, and four studies [8, 22, 23, 25] specifically collected blood samples for the development of the PopPK model. In contrast, retrospective studies primarily used trough samples, as these are commonly used in routine clinical practice. Table **2** summarizes the sampling methods, software used, and validation techniques.

### 3.3 Pharmacokinetic Analysis

Twenty-four vancomycin PopPK models were analyzed: 15 one-compartment models, 6 two-compartment models, and 3 three-compartment models. The structural and statistical data are presented in Table **3**. The most commonly used software was NONMEM [10, 15-17, 19, 20, 22, 23, 26-30], used in 12 out of 22 models, followed by Phoenix NLME [8, 18, 21]. Other software used included Pmetrix [25]^,^ Kinetica [14], Adapt 5 [24], Monolix 4.4 [13, 31], and Pumas 2.0. Internal validation using goodness-of-fit or bootstrap methods, or both, was reported in all studies. Fifteen models also employed advanced internal validation techniques, such as visual predictive checks [13, 16, 17, 19-23, 25-29], numerical predictive checks [15], and/or normalized prediction distribution error [10, 21, 22, 25, 27]. Six studies included external validation using an independent patient cohort, with measures of predictive error, including mean predictive error, absolute predictive error [10, 14, 16, 18, 24, 29], and root mean square error [20].

### 3.4 Estimated Vancomycin Clearance

The mean (SD) vancomycin clearance was 4.23 (2.01) L/h, with a median of 3.73 L/h and a range from 1.19 to 7.64 L/h. Table **3** shows the detailed data for the models and pharmacokinetic parameters, including variability and covariates studied. The type of patient influenced the clearance values. All models developed for neurosurgical patients (Cl = 6.01 ± 0.31 L/h) [11, 27], oncological/hematological patients (Cl = 7.12 ± 0.28 L/h) [13, 16], and ICU patients on ECMO (extracorporeal membrane oxygenation) [23] had Cl values higher than average. The model with the highest Cl was the one developed in pregnant women [9]. ICU models, due to their large variability, were homogeneously distributed across all quartiles.

Age also appeared to influence the clearance models: models with Cl <3.73 L/h had a mean age of 61.2 (4.43) years, while models with Cl greater than the mean had a mean age of 53.8 (7.42) years. Regarding between-subject variability, the highest variability value for Cl was a coefficient of variation of 67.7% in the study by Kanji *et al*. [25], and the lowest was 7% in Zhao et *al*.'s model [17].

### 3.5 Estimated Volume of Distribution

The volume of distribution values exhibited significant variability across the models. In the one-compartment models, the mean (SD) Vd was 66.2 (34.5) L. For the two-compartment models, the mean (SD) central Vd was 36.3 (31.1) L, and the mean (SD) peripheral Vd was 31.8 (17.4) L. The models with the highest Vd values were developed for neurosurgical patients [29], patients undergoing continuous renal replacement therapy (CRRT) [15, 20, 21], and obese patients [8]. In contrast, the models with the lowest Vd values, corresponding to the first quartile, were those for ICU patients on ECMO [23].

The highest coefficient of variation for Vd was 78.27% in the study by Kanji et *al*. [25], while the lowest was 9% in the study by Wang *et al*. [15]. Zhao et *al*.'s model [17] did not report between-subject variability.

### 3.6 Variability of Clearance and Volume of Distribution

The meta-analysis conducted on Cl and the IIV Cl revealed high heterogeneity among the analyzed studies. This was evidenced by the I^2^ statistic, which approached 1 in both cases (0.83 in Cl and 0.98 in IIV Cl), indicating substantial variability across the studies included in the analysis.

**Fig. (2):**
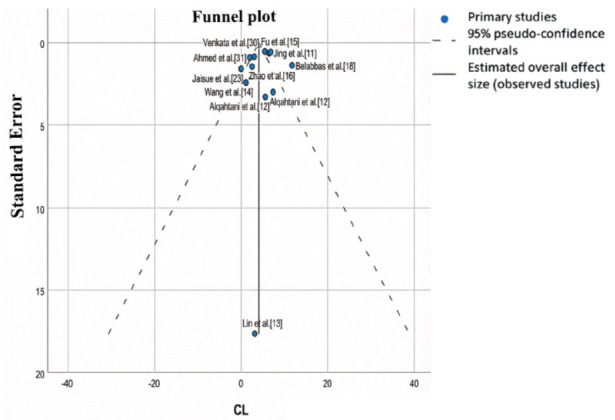
Funnel plot of CL. Funnel plot to assess bias in clearance (CL).

**Fig. (3):**
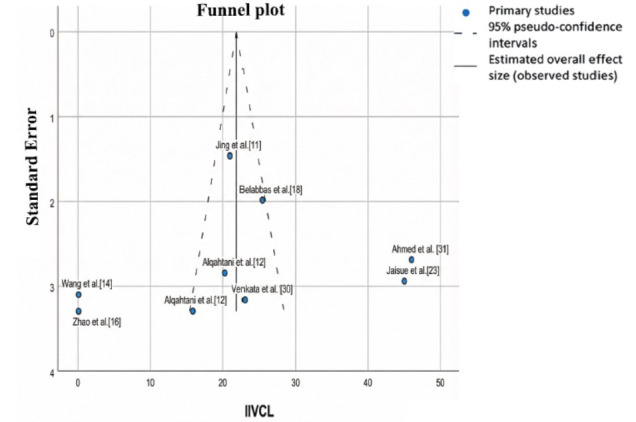
Funnel plot of IIV CL. Funnel plot to assess bias in IIV CL.

Egger’s test confirmed the presence of publication bias in the Cl results (*p*= 0.004), as a strong relationship was observed between the mean Cl value and its dispersion (Fig. **2**). This suggested that Cl results may not be fully generalizable. However, this bias was not present in IIV Cl (Egger’s test: *p*= 0.174), where the relationship between the estimated mean value and its dispersion was weaker (Fig. **3**). This indicates that interindividual variability in clearance was more consistent and generalizable compared to absolute Cl values.

### 3.7 Variables

The statistically significant variables are summarized in Table **3**. These variables included creatinine clearance, weight, ECMO type, SOFA score (sequential organ failure assessment), age, renal dysfunction, nutritional risk, ideal weight, cystatin C clearance, CRRT extraction rate, lean weight, neutropenia, acute myelodysplastic leukaemia, and white blood cell count. ClCr was a significant variable in 66% of the models, and weight was significant in 33% of the models. Residual error was expressed as proportional residual error in 16 models, and as additive residual error in 5 models [15, 19, 27-29]. Three studies presented both types of error [15, 27, 29].

## 4 DISCUSSION

The results of this study have been found to be consistent with previous reviews, such as those conducted by Marsot *et al.* [11] and Aljutayli *et al.* [12], which have also reported high variability among vancomycin pharmacokinetic models. However, this study has uniquely contributed by analyzing more recent models and exploring the inclusion of additional variables that have not been considered in previous studies. The patient populations studied were highly heterogeneous, including those undergoing CRRT, pregnant women, and patients with augmented renal clearance (ARC). Additionally, comorbidities, such as chronic renal failure, diabetes, and critical conditions requiring ICU hospitalization, were documented. The inclusion and exclusion criteria varied among the studies, which may have contributed to the observed variability in pharmacokinetic parameters.

### 4.1 ICU Patients

ICU patients are highly diverse, leading to significant variability in PK parameters across different subpopulations. In this review, we analyzed a total of 12 PK models, including four models of patients undergoing CRRT, one neurosurgical, and one ECMO patient. The development of PK models in this population is challenging due to patient heterogeneity, which affects drug Cl and Vd.

### 4.2 Neurosurgical Patients

Jalusic *et al*. demonstrated that neurosurgical ICU patients have lower vancomycin concentrations, needing higher doses. They identified factors affecting plasma concentrations, highlighting the influence of ClCr and blood-brain barrier permeability as indicated by cerebrospinal fluid (CSF) lactate levels [29]. Continuous infusions of 4 g per day were recommended for these patients. Vancomycin trough levels are generally lower in neurosurgical patients compared to other hospitalized populations [32, 33], leading to reduced efficacy in treating central nervous system infections. The higher Cl in these patients is attributed to clinical factors (stress, vasoactive drugs, drains) [29] and demographics (mean age of 52.7 years *versus* 62 years).

Kim *et al*. [33] performed a comparative study where they found Cl as higher in neurosurgical patients (0.104±0.036 L/h/kg) compared to other ICU patients (0.073±0.042 L/h/kg). Vancomycin, being a high molecular weight molecule, crosses the blood-brain barrier with difficulty, resulting in lower CSF concentrations compared to plasma. CSF vancomycin levels are influenced by meningeal inflammation, with a CSF to plasma concentration ratio of 0.48±0.22 mg/L in patients with meningitis and 0.18±0.05 mg/L in those without [34]. The variability in CSF penetration further complicates achieving therapeutic concentrations, necessitating advanced monitoring and dose adjustments tailored to the inflammatory status of the meninges.

Therefore, studies on intravenous and intraventricular administration have been conducted, albeit with a limited number of patients [29, 35].

### 4.3 ECMO Therapy

ECMO is used for respiratory and/or cardiac failure and involves mixing priming serum with the patient's blood, causing acute hemodilution and reducing drug concentrations [36]. The impact on plasma concentration depends on the drug's Vd, protein binding, and equilibrium between tissue and plasma concentrations [37]. Drugs with small Vd may be significantly affected by hemodilution. Albumin levels, which affect drug binding to plasma proteins, are also impacted, potentially influencing vancomycin’s pharmacokinetics [38].

Vancomycin’s Vd is not significantly affected by ECMO, as confirmed by Donadello *et al*. [37] in their study comparing patients undergoing ECMO *versus* those not undergoing ECMO, finding no significant differences in Vd or Cl. Similarly, Cheng *et al*. [39] did not find significant differences in Vd or Cl in ECMO patients. Other studies’ results [37, 40] have been found to align with the findings of Jung et *al*., indicating that PK model variability often lies in differences in sample sizes and study designs. Jung et *al*. [23] compared two ECMO modalities, venovenous (VV) and venoarterial (VA), and reported significant differences: VV Vd = 0.11 L/kg *vs*. VA Vd = 0.39 L/kg. These Vd differences may be due to the higher proportion of VV ECMO models (13% *versus* 41%).

### 4.4 Impaired Renal Function

We examined three studies on patients undergoing CRRT [15, 20, 21], one on dialysis patients [25], and one on patients with unstable renal clearance [24]. CRRT is common in ICU patients with renal failure and hemodynamic instability. Different CRRT modalities are used, with lower flows in Japan and higher flows in the USA and UK [41]. The Japanese TDM society recommends an initial vancomycin dose of 15-20 mg/kg, followed by 500 mg (7.5-10 mg/kg) every 24 hours as maintenance. Matsumoto *et al*. suggest 500-1500 mg every 24-48 hours for low permeability continuous venovenous hemodialysis and 1000-1500 mg every 24 hours for high permeability continuous venovenous hemodiafiltration to maintain levels of 10-15 mg/L [41]. Doses should be adjusted based on TDM outcomes. Wang et *al*. suggest dosing regimens of 5 mg/kg every 12 hours or 7.5 mg/kg every 12 hours, depending on CRRT intensity. Residual renal function affects vancomycin elimination in CRRT patients [15]. Continuous infusion of vancomycin during CRRT provides more stable levels [42]. Their models showed Cl 1.19 L/h and 1.35 L/h, significantly lower than average [15, 21]. Key covariates identified in CRRT patients included CRRT intensity, reduced urine output, CRRT effluent rate, SOFA score, and weight [15, 20, 21].

### 4.5 Augmented Renal Clearance

ARC, defined as ClCr >130 mL/min [17, 43], increases drug clearance and often results in subtherapeutic vancomycin levels. This condition is prevalent in ICU patients on vasoactive drugs [43], which enhance cardiac output and renal filtration. Key risk factors for augmented renal function [17] include young age, male gender, absence of organ dysfunction, trauma, mechanical ventilation, febrile neutropenia, and high cardiac output [17]. Udy et *al*. created a scale to assess ARC risk, awarding points for age <50 years, trauma, and SOFA score ≤4 [44].

In our review, one model focusing on the ARC population showed a Cl of 5.58 L/h, 1.3 to 2.1 times higher than in the general population [17]. Chu *et al*. [43] reported a Cl of 8.52 L/h, indicating a 2.5 to 3.5-fold increase. In hyperdynamic states, vancomycin distributes more widely, reaching difficult-to-access areas and accumulating more in adipose tissue [44]. Low albumin levels also correlate with higher vancomycin clearance due to an increased free drug fraction that can be renally excreted [43].

### 4.6 Oncohematology Patients

Vancomycin is a key treatment for febrile neutropenia in hematological patients, especially those with acute myeloid leukemia who are at high risk for sepsis. Studies [16, 19, 45] have shown higher Cl in hematological patients, with a mean Cl of 3.47 L/h compared to 2.45 L/h in older patients [16]. Neutropenia has been identified as a significant covariate [19, 45, 46] with Bury et *al*. [45] suggesting a 25% increase in maintenance dose due to a 27% increase in Cl, while Belabbas *et al*. [19] have reported a 17% increase in acute myeloid leukemia patients.

Given the lack of consensus on dose adjustments and the high risk of severe sepsis, guidelines recommend individualized treatment through TDM [45, 47]. Fu *et al.* [16] proposed dosing of 1 g/8 h in hematological patients with high creatinine clearance to quickly reach target levels, without modifying the loading dose since Vd was unaffected.

### 4.7 Obese Patients

Standard vancomycin dosing is 15-20 mg/kg based on total body weight, not exceeding 2 g per administration and 3 g in cases of methicillin-resistant *Staphylococcus aureus* infection [3]. Infusions exceeding 1 g should be administered within 1.5 hours to avoid red man syndrome, with a maximum infusion rate of 15 mg/min. Obese patients are at higher risk of nephrotoxicity due to increased doses and body surface area. Studies suggest potential overdosing, with protocols recommending lower dosages, such as 10 mg/kg instead of 15 mg/kg [8].

Due to variability in this population, efforts to reduce uncertainty include more intensive monitoring by measuring peak and trough levels to estimate the elimination curve. Hong et *al*. [48] and Reynolds et *al*. [49] observed higher overdosing in obese patients and established a dosing protocol of 10 mg/kg instead of 15 mg/kg. Supratherapeutic trough levels may result from drug accumulation in adipose tissue during prolonged treatment [49, 50].

Studies have used different weight metrics, such as ideal body weight and total body weight, which have uncertain impacts on dosing protocols in obese patients [50]. Masich et *al*. [8] developed a nomogram for obese septic patients, adjusting doses based on creatinine clearance to achieve target AUC 24 levels between 400-600 mg·h/L, with doses potentially reaching up to 5 grams.

The limitations of this study included the heterogeneity of the populations analyzed, which posed challenges in determining the suitability of the model. This heterogeneity hindered direct comparisons between models, as no efforts were made to integrate the diverse datasets or validate the models across different contexts. Furthermore, the study was constrained by the limited availability of robust data, inconsistencies in measurement and analysis methods, difficulties in incorporating covariates, and the absence of external validation for the proposed model.

In this regard, the heterogeneity results from the studies identified in the review on Cl and IIV Cl led the authors to dismiss the feasibility of conducting a meta-analysis. However, the selection bias results can be interpreted based on their differing outcomes for the two analyzed parameters. The analysis demonstrated a high variability in Cl values among subjects within the same study, concerning the central parameter analyzed, and revealed a consistent trend across all reviewed studies. Furthermore, considerable variability was detected among patients within the same medical service, introducing uncertainty in the application of predictive models across different hospital centers. On the other hand, interindividual variability exhibited relatively lower dispersion around the central parameter value, implying the obtained values to be more generalizable. Consequently, Cl predictive models cannot be extrapolated between hospitals due to the high heterogeneity of Cl. However, within a single hospital, the application of specific models is feasible, as IIV Cl shows lower dispersion and, therefore, more consistent results within the same clinical setting.

Discrepancies in dosing recommendations also highlighted the need for further validation to ensure the model's applicability in routine clinical practice. These challenges have underscored the need for further research to improve the accuracy and clinical applicability of vancomycin PopPK models.

## CONCLUSION

The findings of this study have important clinical implications. The high variability in vancomycin pharmacokinetic models highlights the need for individualized dosing strategies, reflecting the diverse clinical characteristics of the patient populations studied. In this regard, each hospital should develop its own vancomycin pharmacokinetic models, tailored to the specific characteristics of its patient population. This approach will minimize the observed heterogeneity and improve the accuracy of estimations within each center. The lack of generalizability of models across different hospitals highlights the need for a personalized approach adapted to the clinical and demographic characteristics of each medical institution. Creatinine clearance was a key covariate in most models, emphasizing its role in vancomycin clearance. Other factors, such as weight, age, and specific clinical conditions, like augmented renal clearance and ECMO therapy, also influenced pharmacokinetic parameters. Notably, this is the only study to date that has synthesized pharmacokinetic models published in the last four years, providing a comprehensive overview of recent advancements. Furthermore, it has highlighted underexplored population groups, such as ECMO patients, obese individuals, and neurosurgical patients, whose pharmacokinetics have been less studied until now. The development of vancomycin pharmacokinetic models continues, driven by inter-individual variability and the need to optimize dosing regimens. Future studies should focus on larger, more specific cohorts, integrate additional clinical variables, and explore how these models can be implemented in clinical practice to improve treatment outcomes. In particular, multicenter collaborations, including intensive care units and diverse patient populations, are strongly recommended to ensure external validation and broaden model applicability. Additionally, incorporating clinical decision-support systems and standardized protocols could facilitate real-time integration of these models into routine practice, ultimately enhancing patient safety and therapeutic effectiveness. This synthesis may provide a valuable foundation for understanding recent progress and identifying priorities for future research.

## Figures and Tables

**Table 1:** Anthropometric and demographic characteristics of the patients included in the review.

**Author**	**Country**	**Year**	**Patients**	**Sex (% Male)**	**Age (Years)**	**Weight (Kg)**	**BMI (Kg/m)^2^**	**GFR (mL/min/sup^2^)**	**Characteristics of the Subjects**
Masich *et al*. [8]	USA	2020	16	56%	62 (30-78)	112.7 (72.6-129.1)	36.1 (30.3-45.8)	46 (14-123)	Obese
Kanji *et al.* [25]	Canada	2020	31	71.0%	63.0 ± 17.0	87.4 ± 17.0, (60-138)	29.8 ± 6.3	NR	SLED
Jung *et al.* [23]	Republic of Korea	2021	22	77%	56 (43.3-64.5)	74.0 (55.5-82.3)	-	62.1 (48.9-76.6)	ECMO
Yamazaki *et al*. [21]	Japan	2020	25	68%	65 (21-83)	59 (35.0-92.4)	-	Cr=1.47 (0.23-11.49) mg/dL	CRRT
Huang *et al.*[18]	China	2021	52	75%	65 (49.5-76.5)	65.5 (59.5-72.5)	23.0 (21.5-24.5)	89.1 ± 40.3^a^	PICS
Kovacevic *et al.* [26]	Bosnia and Herzegovina	2020	73	54.8%	56.9 ± 17.0	78.2 ± 14.2	25.7 ± 4.14	80.0 ± 44	NR
Lin *et al.* [14]	China	2021	374	67.9%	62.0 (18.0-93.0)	65.0 (40.0-90.6)	NA	Cr =71 (28.0 -581.0) (µmol/L)	NR
Jaisue *et al.* [24]	Thailand	2020	180	56.7%	60.8 ± 17.5 (17-97)	54.2±11.7 (30.0 -103)	NA	66.2 ± 56.2 (7.3- 281)	NR
Bang *et al.* [22]	Korea	2021	22	68%	67.5 ± 13.2 (36-87)	62.9 ± 10.4 (47.1-90.6)	23.7 ± 3.2 (17.7-29.5)	CKD-EPI=, 101.4 ± 27.9 (52.3-160.9)^a^	NR
Wang *et al.* [15]	China	2022	180	57%	64 (52-72)	85 (73.2-104.7)	NA	NA	CRRT
Jing *et al.* [10]	China	2020	222	76%	46.95 ± 12.71	60.2±11.8	NA	128±35.6	Neurosurgical
Alqahtani *et al.* [13]	Saudi Arabia	2020	73	58%	53.8 ± 15.7	72.7 ± 16.2	27.6 ± 6.4	102 ±58.8	Onco-hematological
			74	60%	55.1 ±15.9	75.5 ± 19.7	27.1 ± 5.8	103 ± 59.3	Non Onco-hematological
Oda *et al.* [20]	Japan	2020	17	58.8%	64 (19-92)	59.7 (43.1-117)	NA	Cr =1.7mg/dL (0.4-7)	CRRT
Aljutayli *et al.* [27]	Canada	2022	116	71%	67.8 ± 11	72 ± 8.6	24.2 ± 3.1	Cr =1.0 ± 0.5 mg/dl	NR
Goyal *et al.* [9]	USA	2022	34	0%	28 (17-38)	74 (43-157)	28 (19-70)	176 (43-389)^b^	Pregnant women
Fu *et al.* [16]	China	2021	77	54%	43.28 ± 15.88 (17- 83)	56.84 ± 11.09 (33.00- 83.50)	21.52±3.63 (14.67-31.87)	118.78 ± 22.69 (45.60- 163.80)^a^	Haematological
Belabbas *et al*. [19]	Japan	2023	148	56.1%	53.6 ± 16.0	58.7 ± 13.1	21.7 ± 3.8	122 ± 52.8 (12.9-324)	Haematological
Munir *et al*. [28]	Pakistan	2021	58	73.7%	54 [25-86]	75 [53-129]	NR	101.15 (15.9-177)	Surgical
Zhao *et al.*[17]	China	2021	209	60.3%	66.0 ± 16.4	63.4 ± 12.9	NR	86.7 (18.4-390.7)	ARC
Jalusic *et al.* [29]	Germany	2021	29	50%	52 (44-61)	80 {70-85 }	NR	152 (109; 174)	EVD
Venkata *et al.* [30]	USA	2024	19	26.3%	31.2± 12.4	63.6 ± 17.1	NR	117 ± 26.3	Cystic fibrosis
Ahmed *et al*. [31]	Saudi Arabia	2024	99	66%	65 (50-75)	NR	NR	12.7 (5.52-25.78)	Renal Impairment

**Note:** Values are expressed as mean ± standard deviation, mean (range) or median [range] or Median {IQR}. GFR (mL/min/sup^2^)=Glomerular filtration rate is obtained using the Cockcroft-Gault equation. a. Glomerular filtration rate obtained using the CKD-EPI equation. If <18 years Schwarz equation^b^.

ARC: augmented renal clearance (ClCr>130ml/min), BMI: body mass index, Cr: serum creatinine, ClCr: creatinine clearance, CRRT: continuous renal replacement therapy, ECMO: extracorporeal membrane oxygenation, EVD: external ventricular drain, ICU: intensive care unit, NR: not reported, PICS: immunosuppression and catabolism syndrome, SLED: Sustained low-efficiency dialysis.

**Table 2:** Sampling details and study design.

**Author**	**Number Total Samples**	**Steady State**	**Sampling frequency**	**Type of Study**	**Source of Data**	**Software**	**Type of Validation**
Masich *et al*. [24]	64	No	peak, trough and two ramdoms	Prospective	PK study	Phoenix NLME v.8.1	Basic internal: GOF, bootstrap (n=500)
Kanji *et al*. [25]	335	NR	During the session: Trough and 1, 3 and 5 hours After session 1, 3 and 5 h	Prospective	PK study	Pmetrics	Basic internal: GOF, bootstrap (n=1000). Advanced internal: NPDEs, VPC.
Jung *et al.* [22]	194	NR	ClCr≥50 ml/min/1.73 m3 : Trough 0, 1, 1.5, 2, 3, 4, 4, 6, 8 and 12 h ClCr<50 ml/min/1.73 m3 0, 1, 1.5, 2, 3, 3, 4, 10, 16, and 24 h.	Prospective	PK study	NONMEM 7.4,	Basic internal: GOF, bootstrap (N=2000) Advanced internal: VPC.
Yamazaki *et al.* [20].	130	NR	Trough and random	Retrospective	Routine TDM	Phoenix NLME 1.4, WinNonlin 6.4.	Basic internal GOF, bootstrap (n=1000) Advanced internal: VPC, NPDE.
Huang *et al.* [17]	126	YES	Trough	Retrospective	Routine TDM	Phoenix NLME 1.4	Basic internal GOF, Bootstrap (n=500). External validation: MPE, MAE.
Kovacevic *et al*. [26]	146	YES	Peak / trough	Prospective	Routine TDM	NONMEM^®^ 7.3	Basic internal:GOF, bootstrap (n=1000) Advanced internal: VPC.
Lin *et al.* [13]	993	YES	Peak (156) / trough (837)	Prospective	Routine TDM	Kinetica 4.4.1	Basic internal: GOF. External validation: MPE, MAE.
Jaisue *et al.* [23]	474	NR	Trough	Retrospective	Routine TDM	ADAPT 5	Basic internal: GOF. External validation: MPE, MAE.
Bang *et al*. [21]	197	NO	Doses less than 1.5 g dose: 0, 20, 40, 60, 60, 80 min, 2, 6, 12, 24 h; Doses greater than 1.5 g: 0, 30, 60, 90, 90, 110 min, 3, 6, 12, 24 h.	Prospective	PK study	NONMEM 7	Basic internal: GOF, bootstrap (n=2000). Advanced internal: VPC, NPDE.
Wang *et al*. [14]	1186	YES	During CRRT	Retrospective	Routine TDM	NONMEM 7.2.0	Basic internal: GOF, boostrap (n=1000). Advanced internal: NPC.
Jing *et al*. [10]	436	YES	Trough mostly	Retrospective	Routine TDM	NONMEM 7.4.3	Basic internal: GOF, bootstrap (n=2000). Advanced internal: NPDE. External validation: MPE, MAE
Alqahtani *et al*. [12]	448	YES	Trough	Retrospective	Routine TDM	Monolix 4.4	Basic internal: GOF, bootstrap (n=1000). Advanced internal: VPC.
Oda *et al.* [19]	80	YES	Trough	Retrospective	Routine TDM	NONMEM 7.3	Basic internal: bootstrap (n=1000) Advanced internal VPC External: RMSPE
Aljutayli *et al.* [28]	326	YES	Peak / trough	Retrospective	Routine TDM	NONMEM 7.4	Basic internal: bootstrap (n=1000) Advanced internal VPC and NPDE
Goyal *et al*. [30]	91	YES	Trough mostly	Retrospective	Routine TDM	Pumas 2.0	Basic internal: GOF, bootstrap (n=1000)
Fu *et al.* [15]	152	YES	Trough mostly	Retrospective	Routine TDM	NONMEM	Basic internal: GOF, bootstrap (n=1000). Advanced internal: VPC External validation: MPE, MAE
Belabbas *et al*. [18]	681	YES	Trough	Retrospective	Routine TDM	NONMEM^®^ 7.4.3	Basic internal: GOF, bootstrap (n=1000) Advanced internal: VPC
Munir *et al.* [29]	176	NO (first dose)	NR	Prospective	NR	NONMEM 7.4.4)	Basic internal: GOF, bootstrap (n=1000). Advanced internal: VPC
Zhao *et al*. [16]	424	NR	NR	Retrospective	Routine TDM	NONMEM 7.7	Basic internal: GOF, bootstrap (n=1000). Advanced internal: VPC
Jalusic *et al*. [27]	184 blood, 133 CSF	NR	Trough	Retrospective	Routine TDM	NONMEM 7.3	Basic internal: GOF, bootstrap (n=1000). Advanced internal: VPC External validation: MPE, MAE
Venkata *et al.* [30]	181	Yes	Peak / trough	Retrospective	Routine TDM	NONMEM 7.5	Basic internal: GOF, Advanced internal: VPC
Ahmed *et al.* [31]	194	Yes	Peak / trough	Retrospective	Routine TDM	MonolixSuite 2020R1.	Basic internal: GOF, Advanced internal: VPC External validation: NPDE, boostrap

**Note:** ClCr: Creatinine clearance, CRRT: renal replacement therapy, CSF: cerebrospinal fluid, GOF: Goodness-of-fit, MAE: absolute predictive error, MPE: mean predictive error, NPC: numerical predictive check, NPDE: normalized prediction distribution errors, PK: Pharmacokinetics, RMSPE: root mean square error, TDM: Therapeutic drug monitoring, VPC: visual predictive check.

**Table 3:** Pharmacokinetic models and parameter variability.

**Author**	**Compartments**	**Pharmacokinetic Parameters**	**Variability Model**	**Significant Covariates**
Masich *et al*. [24]	One compartment	Cl= 3.23 L/h (CSR=7.11%) Vd= 85.0 L (RSE=5.42%)	BSV Cl=31.5% BSV Vc=12.4% Prop REE=8.89%	Clcr
Kanji *et al*. [25]	Two compartments	Cl_SLED_: 5.97 L/h (CSR=67.75%) Cl: 2.40 L/h (RSE=60.64%) ClSLED _ON=_ 8.37 L/h ClSLED _OFF=2_.40 L/h V1= 29.56 L (RSE=78.27%) V2= 26.50 L (RSE=43.34%) K_1-2_ =10.84 h^-1^ (CSR =57.61%) K_2-1_ =12.09 h^-1^ (RSE =104.04%)	BSV Cl=67.75% BSV Vc=78.27%	SLED
Jung *et al.* [22]	Three compartments	Cl = 4.01 L/h (RSE=7.51%) V1 =8.01 L (RSE=8.43%) V2=15.4 L (RSE=8.53%) V3= 6.21L (RSE=14%) Vd=29.6 L Q_V1-V2_: 4.95 L/h (5.27% for VA ECMO, 6.23 L/h (17.1%) for VV ECMO.	BSV Cl=33.9%(RSE 13.7%) BSV V1=27.9% (RSE =18.3%) BSV V2=34.3% (RSE =23.3%) BSV V3=56.9% (RSE =23.3%) Prop REE=6.64% (RSE=15.5%)	GFR CKD.EPI, ECMO type
Yamazaki *et al.* [20]	Two compartments	Cl =1.35 L/h (RSE=8.7%) V1=59.10L (RSE=9.2%) V2= 56.14L (RSE=12.8%) Q_V1-V2_ =3.65L/h (RSE=13.8%)	BSV Cl=NA BSV V1=33.4% (RSE=11.8%) BSV V2=112.3% (RSE=17.5%) BSV Q _V1-V2_ =9.7% (RSE=6.8%) Prop REE=16.40% (RSE= 9.3%)	SOFA, BW
Huang *et al*. [17]	One compartment	Cl =3.03 (RSE=8.41%) V1= 42.4 L (RSE=NA)	BSV Cl=28.5% (NA) Prop REE=0.288% (RSE=10.5%)	CKD-EPI cys-cr
Kovacevic *et al*. [26]	One compartment	Cl=3.73 L/h V1=39.96 L	IIVCl: 56.6%. Prop REE=34.5% Prop REE=34.5%	Clcr
Lin *et al.* [13]	One compartment	Cl 3.16 L/h (RSE=56.01%) V1=60.71L (RSE=55.19%)	IIV Cl:41.72 IIV V1:35.14% REE=23.54%	ClCr, age, BW
Jaisue *et al.* [23]	Two compartments	SClr =0.021 L/h/mL/min (RSE= 7.20%) ClNR (non-renal) =0.11 L/h (RSE=23.7%) V1=13.8L (RSE= 21.3%) V2= 44.7 L (RSE=9.02%) Q_V1-V2_ =3.54L/h (RSE=26.3%)	IIV SClR = 45% (RSE: 13.5%) IIV ClNR= 17.2% (RSE: 124%) IIV V1 =47.5% (RSE: 35.3%) IIV V2= 47.6% (RSE: 14.7%) IIV Q= 58.6% (RSE: 58.0%) PropREE = 17.6% (RSE: 4.91%)	CrCl
Bang *et al*. [21]	Three compartments	𝜃Cl\_Age > 65= 0.0143 (RSE=44.1%) 𝜃Cl\_Age ≤ 65= 0.0349 (RSE=23.5%) Cl\_wt=0.0271 (CSR=26.5%) θ V1=4.04 (CSR=10.8%) θV1_IBW=4.21 (RSE=32.3%) θ V2=16.6 (RSE=8.3%) θ V3=42.6 (RSE=10.2%) θV3_IBW=12.7 (RSE=9.7%) Q1(L∕min) = 0.294 (RSE=6.8%) Q2 (L∕min) = 0.0816 (RSE=13.0%)	IIV CL age>65 - CV%: 21.3%. IIV V1: 15.5%. IIV V2: NA% NA% IIV V2: NA% IIV V2: NA% IIV V2: NA IIV V3: 11.1%. IIV Q1: 18.4%. IIV Q 2: 33.2%. REE %: 39% (RSE=13%)	Age > 65 years: Ideal BW, BW, age. Age ≤ 65 years: Ideal BW
Wang *et al*. [14]	One compartment	CL_POP_ = 1.19 L/h (RSE=7%) V_POP_ = 107 L (RSE=5%) α=0.797 (RSE=29%) β=0.0325 (RSE=11%)	IIV CL =0.0938%(RSE=9%) IIV Vc = 0.255 (RSE=9%) Prop REE=0.0476 (RSE=6%) Add REE=3.33 (RSE= 29%)	CRRT intensity
Jing *et al*. [10]	One compartment	Cl SCR=6.49 L/h (RSE=3%) V1SCR=60.2L (RSE=9%)	IIV CLSCR=21% (RSE=7%) IIV Vc SCR = NA cysC: 6.3% (RSE=9%)	sClCr, age.
	One compartment	ClcysC 6.40 L/h (3%) V1 cysC=62.00 L (9%)	IIV CLcysC=20.40% (RSE=7%) IIV V1 cysC= NA Residual variability cysC: 6.5% (RSE=9%)	ClCr, age weight
Alqahtani *et al*. [12]	*Cancer patients* One compartment	Cl=7.4 L/h (RSE=20%) V1 =45 L (RSE=15%)	IIV Cl=15.9% (RSE=22%) IIV V1=13.8% (RSE=12%) REEonco= 0.125 (RSE=7%)	Clcr
	*No cancer patients* One compartment	Cl = 5.6 L/h (RSE=22%) Vc no onco = 42 L (RSE=13%)	IIV Cl=20.3% (19%) IIV V1 =18.2% (RSE=20%) REE no onco= 0.23 (RSE=12%)	
Oda *et al.* [19]	One compartment	Cl_NONCRRT=2_.12 L/h V1=91.3L/70kg θ1= 2.12 (RSE=10.8%) θ2 =16 (RSE=10.3%) θ3 =0.672 FIXED^a^ θ4= 91.3 (RSE=13.9%)	BSV Cl=30.4% (RSE =30.0%) BSV V1=35.3% (RSE =45.4%) Prop REE=15.7% (RSE= 29.3%)	RUO and CL _CRRT_
Aljutayli *et al.* [28]	One compartment	θ 1= 4.16 L/h (RSE=4.1%) θ 2= 102.46L (RSE=9.7%)	BSV Cl=34.12% (RSE 11.2%) BSV V1=51.83% (RSE=16.8%) Prop REE=13.95% (RSE=29.9%) Add REE=3.04mg/L RSE=19.7%)	Clcr, WT
Goyal *et al*. [30]	Two compartments	Cl (L/h) = 7.64 L/h V1 (L)= 67.35L V2 (L)= 37.5L Q (L/h)= 9.06 L/h	IIV Cl=31.9% IIV Prop REE=42.1% (RSE =0.21%)	ClCr, FFM
Fu *et al.* [15]	Two compartments	Cl =6.84 L/h (RSE=4.5%) V1=20.5 L (RSE=17.3) V2= 50 L (RSE=27.2%) Q_V1-V2_ =15.2 L/h (RSE=23.4%)	IIV CL =0.0615% IIV Vd =0.126 η1 =17.8%(RSE=15%) η2=33% (RSE=35.8%)	BW, ClCr
Belabbas *et al*. [18]	One compartment	Cl =3.09L/h (RSE=3.2%) V1=122 L/70kg (RSE=7.1%)	IIV Cl =25.4% (RSE=7.6%) IIV V1=47.4% (RSE=14.2%)	ClCr, BW, neutropenia, AML
Munir *et al.* [29]	One compartment	Cl =2.45 L/h (RSE=2.0%) Cl-CRCL =0.0046 (RSE =13%) Cl-WT =0.011 (RSE =10%) V1 (L)=22.6 (RSE=5%)	BSV Cl =11.3% (RSE= 38%) Add REE=3.07 (RSE=10%)	BW, ClCr
Zhao *et al*. [16]	Two compartments	Cl =2.56 L/h (RSE=7%) Cl max = 5.58 L/h(RSE= 17%) CG Cl max50 (L/h)=93.8 (RSE=24%) V1=13.1 L (RSE=11%) V2= 40.2 L (RSE=13%) Q_Vc-Vp_ =4.9L/h(RSE=26%)	IIV CL= 0.0771 (RSE=16%) IIV Vc = 0.223 (RSE=14%) Add REE=0.0479% (RSE=18%)	ClCr
Jalusic *et al*. [27]	Three compartments	Cl =5.15L/h (RSE =NA%) V1=41.13 L (RSE =NA%) V2= 0.32L (RSE=NA%) V3= 86.2L (RSE=NA%) Q =3.61L/h (RSE=NA%) CL_dif_ =0.0031 L/h (RSE=NA%)	IIVCl=34.9%(RSE=NA%) IIV Cl_dif_ =65.4%(RSE=NA%) Prop REE=18.5% (RSE=NA%) Add REE=3.16% (RSE=NA%)	ClCr CSF-lactate
Venkata *et al.* [30]	One compartment	Cl= 5.5 L/h/70kg (RSE=3.8%) V1=31.5 L/70Kg (RSE=4.6%)	IIVCl =23% (RSE=23.5%) RUV = 0.0413 (12.6%)	Weight
Ahmed *et al.* [31]	One compartment	Cl= 2.02 L/h/70kg (RSE=6.4%) V1=65 L (RSE=9.41%)	IIVCl =0.46% (RSE=0.061%) IIV =0.39% (RSE=19.3%) Prop Ree = 0.28 (8.26%)	ClCr

**Note:** Add REE: Additive relative error, AML: acute myeloid leukemia, ARC: augmented renal clearance, BSV: Between-subject variability, BW: Body weight, CE: glomerular filtration rate obtained by the CKD-EPI equation, CKD-EPI cys-cr: glomerular filtration rate obtained using the CKD-EPI equation and serum cystatin C concentration. Cl: vancomycin clearance. ClCr: Creatinine clearance, CSF:cerebrospinal fluid barrier, CV: coefficient of variation, DA: dopamine, ECMO: extracorporeal membrane oxygenation, ECMO-VA, venoarterial ECMO; ECMO-VV, venovenous ECMO, IIV: inter-individual variability expressed by CV= coefficient of variation, FFM: fat free mass, K_1-2_: distribution constant from central to peripheral compartment, K_2-1_: distribution constant from peripheral to central compartment, η reflects the interindividual variability, a random probability distribution of the variability of each subject with respect to the population of type N(0,ω2), NON SLED: No treatment with sustained low efficiency dialysis, ON SLED: during sustained low efficiency dialysis, OFF SLED: Not during sustained low efficiency dialysis, Prop REE: Proportional relative error, RUV =Residual unexplained variability, Q= Vancomycin clearance between central and peripheral compartment, renal replacement, RSE: residual standard error, RUO: reduced urine output, SOFA score: Sequential Organ Failure Assessment, V1: Volume of distribution in the central compartment, V2: Volume of distribution in the peripheral compartment. V3: volume of distribution in the deep compartiment.

## Data Availability

The data that support the findings of this study will be available from the corresponding author upon reasonable request.

## LIST OF ABBREVIATIONS

AJM: Ángela Jimeno-Martín
ARC: Augmented Renal Clearance
AUC: Area Under the Curve
AUC/MIC24: Ratio of the 24-hour Area Under the Curve to the Minimum Inhibitory Concentration
Cl: Clearance
ClCr: Creatinine Clearance
CRRT: Continuous Renal Replacement Therapy
CSF: Cerebrospinal Fluid
ECMO: Extracorporeal Membrane Oxygenation;
ICU: Intensive Care UNIT
IIV: Interindividual variability
INP: Irene Navarro-Pardo
LS-BS: Loreto Sáez-Benito-Suescun
LSC: Lucía Sopena-Carrera
MAAB: María de los Ángeles Allende-Bandrés
MAM: Mercedes Arenere-Mendoza
MGB: Manuel Gómez-Barrera
MIC: Minimum Inhibitory Concentration
NBT: Nuria Berenguer-Torrijo
PK: Pharmacokinetics
PK/PD: Pharmacokinetics/Pharmacodynamics
PopPK: Population Pharmacokinetics
RFM: Raquel Fresquet-Molina
SD: Standard Deviation
SOFA: Sequential Organ Failure Assessment
TDM: Therapeutic Drug Monitoring
TSG: Tránsito Salvador-Gómez
VA ECMO: Venoarterial Extracorporeal Membrane Oxygenation
Vd: Volume of Distribution

## STANDARD OF REPORTING

PRISMA guidelines were followed.

## SUPPLEMENTARY MATERIAL

PRISMA checklist is available as supplementary material on the publisher’s website along with the published article.
